# Maladie de Castleman: localisation inhabituelle du thorax

**Published:** 2012-08-22

**Authors:** Fayçal Eloueriachi, Mohammed Caidi, Fouad Zouaidia, Fahd Ouchen, Mehdi Maidi, Hicham Fennane, Mohamed Bouchikh, Abdellah Achir, Abdellatif Benosman

**Affiliations:** 1 Service de chirurgie thoracique, Hôpital Avicenne, CHU Rabat, Maroc; 2 Service d'anatomie pathologique, Hôpital Avicenne, CHU Rabat, Maroc

**Keywords:** Maladie de Castleman, localisation, thorax, exérèse, chirurgie, Castleman disease, location, chest, excision, surgery

## Abstract

La maladie de Castleman est une affection rare qui peut toucher le thorax. La localisation diaphragmatique est exceptionnelle. Nous rapportons le cas d'une patiente de 47 ans, chez qui une thoracotomie exploratrice a permis l'exérèse d'une masse du sinus médiastinal antérieur droit, en continuité avec le diaphragme et dont l'histologie est en faveur de la maladie de Castleman de type hyalino-vasculaire. Les particularités cliniques, radiologiques et évolutives ont été revues.

## Introduction

La maladie de Castleman dont le nom est attribué à Benjamin Castleman a été décrite pour la première fois par cet anatomopathologiste Américain en 1954 [1]. Il s'agit d'un trouble du tissu lymphoïde caractérisé par une hyperplasie lymphoïde qui peut exister sous deux formes: hyalino-vasculaire; la plus fréquente, et plasmocytaire (10%), une forme mixte a été décrite dont la prévalence reste très rare [2]. Cliniquement la maladie de Castleman peut se développer sous deux formes : l'une localisée, bénigne de découverte souvent fortuite, et l'autre multicentrique de pronostic généralement réservé. La majorité des cas se développent le long des chaines ganglionnaires du médiastin, surtout de l'arbre trachéobronchique et des hiles pulmonaires. Certaines formes extrathoraciques ont été décrites surtout au niveau du rétro péritoine, du pelvis, des aires ganglionnaires périphériques et dans la région cervicale. Nous rapportons le cas d'une localisation intrathoracique inhabituelle de la maladie de Castleman au niveau de l'angle cardiophrénique avec revue de la littérature.

## Patient et observation

**Figure 1:**
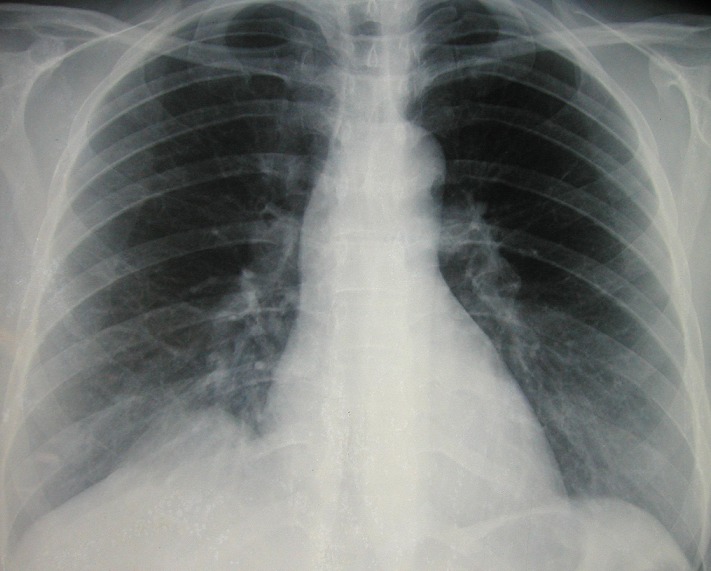
Radiographie thoracique de face montrant une opacité basale paracardiaque droite se confondant avec le diaphragme

**Figure 2:**
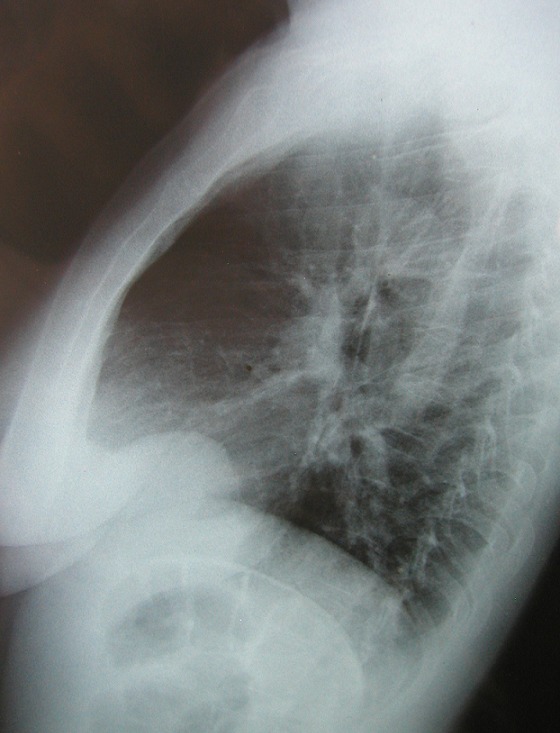
Radiographie thoracique de profile montrant l'aspect d'une surélévation localisée en avant de la coupole diaphragmatique

Madame L.T. est une patiente de 47 ans qui nous a été adressée en septembre 2009 après la découverte sur une radiographie pulmonaire systématique d'embauche d'une opacité basale paracardiaque droite donnant un aspect de surélévation localisée de la coupole diaphragmatique droite (Figure 1), bien visible dans sa partie antérieure sur la radiographie thoracique de profil (Figure 2). La patiente était asymptomatique et ne présentait aucun antécédent pathologique particulier. L'examen somatique, en particulier celui des aires ganglionnaires périphériques était normal.

**Figure 3:**
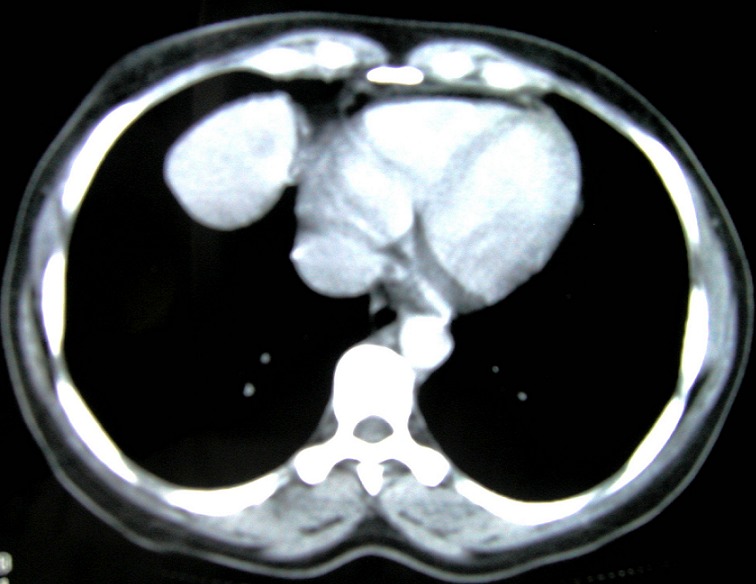
Coupe scannographique objectivant une masse tissulaire bien limitée au contact du péricarde

L’échographie abdominale avec balayage thoracique avait objectivé une masse anéchogène latérocardiaque droite au contact du diaphragme, mesurant 6x5 cm, évoquant une structure graisseuse ou kystique. La tomodensitométrie thoraco-abdominale avait révélé la présence d'une masse mesurant 55 mm de grand axe, de structure mixte : lipomateuse et liquidienne, bien limitée, qui s'est rehaussée après injection de produit de contraste, se projetant sur la partie antérieure du diaphragme et arrivant au contact du péricarde, sans anomalies du parenchyme pulmonaire, du médiastin ou à l’étage abdominal (Figure 3).

Les analyses biologiques ont montré une numération de formule sanguine normale, l'absence de syndrome inflammatoire et une sérologie hydatique négative. A ce stade, les diagnostics différentiels de cette lésion étaient essentiellement : un kyste diaphragmatique congénital, un lymphome, un lipome pleural ou un mésothéliome. Une thoracotomie exploratrice passant par le 6^ème^ espace intercostal droit a été réalisée retrouvant une masse ferme richement vascularisée d'environ 6 cm de grand axe, en continuité avec le diaphragme et siégeant au niveau du sinus médiastinal antérieur droit. Cette masse a été libérée du diaphragme sans effraction de ce dernier, puis fut extraite après ligature-section de son pédicule. Les suites opératoires étaient simples, et le séjour hospitalier était de 4 jours.

**Figure 4:**
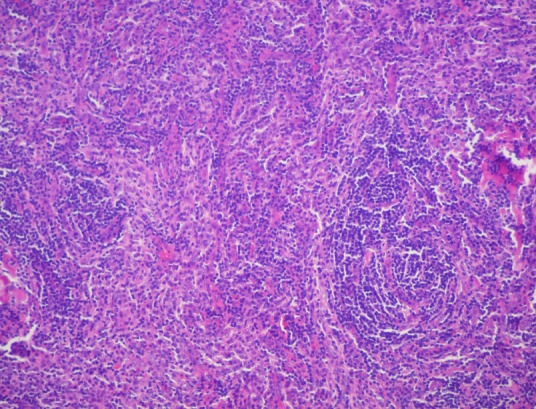
Examen histologique montrant un follicule lymphoïde atrophique HE Gx10

**Figure 5:**
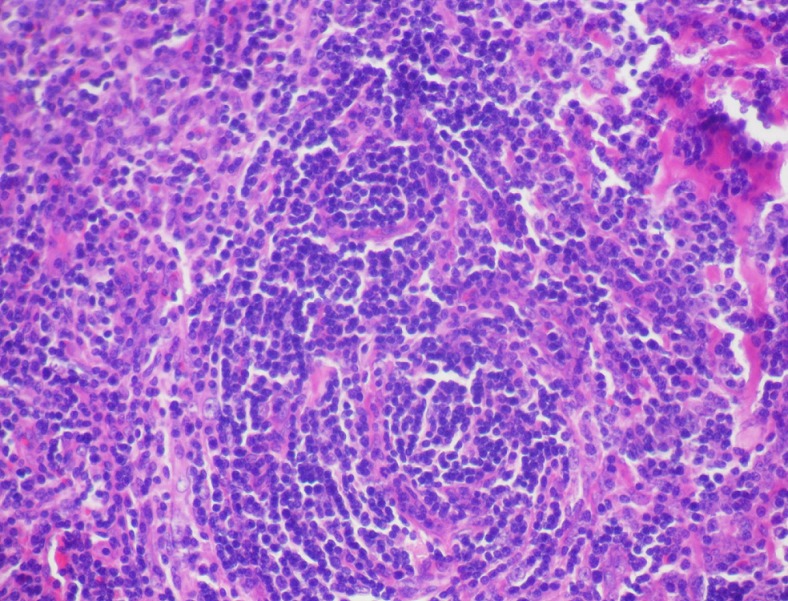
Coupe histologique objectivant la présence de cellules lymphoïdes disposées en file indienne HE Gx40

L’étude macroscopique a objectivé une masse pesant 20 g, avec à la coupe un aspect jaunâtre, dure, homogène contenant quelques remaniements hémorragiques. L’étude histologique a montré une structure ganglionnaire caractérisée par une hyperplasie folliculaire avec atrophie des centres germinatifs. La zone interfolliculaire est très élargie caractérisée par une hyperplasie angioïde avec par endroits des parois vasculaires hyalinisées, associée à un infiltrat principalement lymphocytaire comprenant de nombreux plasmocytes. Cette description étant caractéristique de la maladie de Castleman de type hyalino-vasculaire (Figure 4, Figure 5). Le suivi clinique et radiologique étalé sur une période de 10 mois n'a révélé aucune récidive et la patiente demeure asymptomatique.

## Discussion

La maladie de Castleman décrite comme une hyperplasie lymphoganglionnaire est une affection relativement rare, dont le siège électif reste le médiastin. En effet c'est une affection qui peut se développer là où les ganglions lymphatiques existent, surtout aux dépends des chaines ganglionnaires du médiastin avec une fréquence élevée le long de l'arbre trachéobronchique et des hiles pulmonaires. Des cas rares de maladie de Castleman développés aux dépends des tissus mous de la paroi thoracique (muscles intercostaux et diaphragme) ont été rapportés [3, 4].

L’étiopathogénie de cette affection reste inconnue, quoique plusieurs hypothèses ont été avancées, essentiellement en cas de maladie de Castleman multicentrique, impliquant : des phénomènes inflammatoires chroniques avec dérèglement de la production d'interleukine 6 [5], l'immunodépression liée à l'infection par le virus d'immunodéficience humaine (HIV), et enfin une réponse immunitaire atypique à l'infection par le HHV8 (Human Herpes Virus 8) [6].

La maladie de Castleman localisée demeure durant longtemps asymptomatique, elle est de découverte souvent fortuite. Son expression clinique est toujours en rapport avec une compression des structures adjacentes surtout lorsque la tumeur atteint des volumes importants, donnant : une toux, une dyspnée, des douleurs thoraciques voir une infection bronchique. La localisation phrénique antérieure dans notre cas explique l'absence des signes cliniques habituellement retrouvés.

La radiographie standard objective cette affection dans sa forme localisée sous forme le plus souvent d'une opacité, bien limitée, homogène, parfois avec des calcifications centrales. A ce stade de diagnostic, une hernie diaphragmatique localisée était le premier diagnostic évoqué dans notre cas. Le scanner thoracique montre généralement une masse homogène, tissulaire qui se rehausse après injection de produit de contraste essentiellement dans sa forme hyalino-vasculaire [7]. Dans sa forme multicentrique, l'aspect tomodensitométrique est celui d'une atteinte ganglionnaire médiastinale et hilaire avec présence parfois d'opacités nodulaires parenchymateuses. L'imagerie par résonnance magnétique [IRM] réalisée dans certains cas rapportés dans la littérature ne révèle aucun signe spécifique à la maladie.

Les caractéristiques radiologiques de la maladie de Castleman permettent difficilement d’évoquer le diagnostic, et le recours aux moyens d'investigation invasifs s'avère toujours nécessaire pour asseoir le diagnostic positif.

Le recours à la biopsie scannoguidée permet rarement de poser le diagnostic de certitude ; elle montre le plus souvent un infiltrat lymphocytaire dont la nature lymphomateuse ne peut être écartée. L'exérèse chirurgicale reste dans la majorité des cas le meilleur moyen pour assurer le diagnostic positif et permettre un geste curatif. L'exérèse radicale est la règle quoique difficile en cas de masses intimement adhérentes aux structures de voisinage. Des cas de récidives ont été rarement rapportés surtout après résection subtotale, dans ce cas la radiothérapie a été décrite comme alternative [8].

La chirurgie thoracoscopique vidéoassistée (VATS) a été rapportée comme étant une technique moins invasive avec une efficacité comparable à la thoracotomie dans le diagnostic et le traitement d'une grande variété de lésions thoraciques, surtout s'il n'existe pas d'envahissement des structures adjacentes et que la taille de la lésion est inférieure à 5 cm. Des cas de maladie de Castleman réséqués avec succès par VATS ont été rapportés [9, 10]. La conversion d'une VATS en une thoracotomie reste envisageable devant le risque éminent de saignement que présente ce genre de lésions richement vascularisées.

## Conclusion

La localisation diaphragmatique de la maladie de Castleman est très rare. La présentation clinico-radiologique n'est pas spécifique. L'exérèse radicale reste le meilleur moyen pour asseoir le diagnostic et apporter une satisfaction quant à l’évolution favorable sans récidive de cette affection.
